# Implementing a Health Care Professional–Supported Digital Intervention for Survivors of Cancer in Primary Care: Qualitative Process Evaluation of the Renewed Intervention

**DOI:** 10.2196/36364

**Published:** 2022-04-01

**Authors:** Jazzine Smith, Rosie Essery, Lucy Yardley, Alison Richardson, Joanna Slodkowska-Barabasz, Claire Foster, Eila Watson, Chloe Grimmett, Adam W A Geraghty, Paul Little, Katherine Bradbury

**Affiliations:** 1 Clinical and Community Applications of Health Psychology School of Psychology University of Southampton Southampton United Kingdom; 2 School of Psychological Science University of Bristol Bristol United Kingdom; 3 School of Health Sciences University of Southampton Southampton United Kingdom; 4 Cancer Care Group University Hospital Southampton Southampton United Kingdom; 5 Community and Public Health Faculty of Health and Life Sciences Oxford Brookes University Oxford United Kingdom; 6 Primary Care Research University of Southampton Southampton United Kingdom

**Keywords:** process evaluation, digital intervention, primary care, health care professional, web-based, quality of life, posttreatment, oncology

## Abstract

**Background:**

Primary care plays an important role in supporting survivors of cancer; however, support is limited because of practitioners’ perceived lack of expertise and time. A digital intervention for survivors of cancer could provide an efficient way for primary care staff to support survivors of cancer without the need to accumulate expertise and skills to help patients make behavior changes; providing very brief support alongside this could maximize adherence to digital interventions. *Renewed* is a digital intervention that combines web-based behavior change advice with brief health care practitioner support from a nurse or health care assistant. Knowledge about the views and experiences of primary care staff providing support alongside a digital intervention for survivors of cancer is sparse, limiting the understanding of the acceptability and feasibility of this type of intervention.

**Objective:**

This study aims to explore supporters’ experiences of providing support to survivors of cancer using *Renewed*, understand potential barriers to and facilitators of the implementation of *Renewed* in practice, and investigate the strengths and weaknesses of the intervention from the perspective of health care professionals.

**Methods:**

This was a qualitative process evaluation nested within a large trial evaluating *Renewed*. A total of 28 semistructured telephone interviews were conducted with nurses and health care assistants. Data were analyzed using inductive thematic analysis.

**Results:**

Four themes were developed during the analysis, which reflected the factors that supporters identified as hindering or enabling them to provide support alongside *Renewed Online*: *Renewed Online* as an acceptable digital tool with some improvements, confidence in enacting the supporter role, practicalities of delivering support alongside a digital intervention, and managing a patient-led approach. The analysis suggests that supporters perceived that a digital intervention such as *Renewed* would be beneficial in supporting survivors of cancer in primary care and fit within current practices. However, barriers to providing support alongside the intervention were also identified, including concerns about how to facilitate rapport building and, in a minority, concerns about using a nondirective approach, in which most advice and support is provided through digital interventions, with brief additional support provided by primary care staff.

**Conclusions:**

These findings add to the literature on how best to provide support alongside digital interventions, suggesting that although most practitioners cope well with a nondirective approach, a minority requires more training to feel confident in implementing this. This study suggests that the barriers to providing formal support to survivors of cancer in primary care could be successfully overcome with an approach such as *Renewed*, where a digital intervention provides most of the support and expertise, and health care practitioners provide additional brief human support to maximize engagement. Strategies to maximize the chances of successful implementation for this type of intervention are also discussed.

## Introduction

### Background

In 2018, the total number of people alive within 5 years of a cancer diagnosis was estimated to be 43.8 million worldwide [1]. Currently, there are 2.5 million survivors in the United Kingdom, which is estimated to increase to 4 million by 2030 [2]. However, up to 86% of people who complete cancer treatment in the United Kingdom, Australia, and the United States experience enduring side effects [3-5], including fear of cancer recurrence, anxiety, depression, fatigue, and weight gain, contributing to a reduced quality of life (QoL) [4].

The rising cancer burden places a strain on health systems worldwide [6]. Health care professionals (HCPs) based in primary care are central to providing support for people who have had cancer after completion of their primary treatment (eg, chemotherapy). However, these services are becoming overstretched and are increasingly unable to meet the needs of survivors of cancer [7]. For instance, survivors of cancer have expressed a need for more support with the emotional effects of cancer and issues such as fatigue that can occur months or years after treatment [8]. Primary care staff describe a lack of clear guidance on how survivors of cancer should be supported [9]. Patients and oncologists have expressed concerns that primary care staff are not experts, and their busy workloads lead to deficiencies in the continuity of care [8,9], meaning that survivors of cancer may not receive access to appropriate support with their ongoing symptoms after cancer treatment. Therefore, there is a need for clearer, more effective, and cost-efficient means of providing support. Digital interventions, such as websites or mobile apps, offer the potential to help survivors of cancer improve their QoL [10]. The addition of brief human support can boost engagement with digital interventions [11,12]. Digital interventions combined with brief support from primary care staff may facilitate improved QoL after cancer treatment. It may provide efficient and low-cost models for delivering support without the need to accumulate expertise in the skills and knowledge needed to help patients make the behavioral changes needed to increase their QoL. However, the acceptability and feasibility of implementing digital interventions among survivors of cancer in primary care is still to be determined. An important aspect of this involves understanding the capability of HCPs to deliver brief support along with digital interventions.

*Renewed* [13-16] is a complex intervention designed to improve the QoL of survivors of cancer. It combines a digital intervention focused on changing key behaviors that can improve the QoL of survivors of cancer with brief support from a nurse or health care assistant to maximize engagement. *Renewed* was designed for implementation in primary care within the United Kingdom’s National Health Service (NHS). *Renewed* is currently being tested in a randomized controlled trial (RCT) to determine its effectiveness and cost-effectiveness. In addition to determining the effectiveness and cost-effectiveness of an RCT, it is critical to examine whether an intervention might be implemented well in practice. Understanding barriers to and facilitators of implementation could help optimize the implementation of *Renewed Online* and also provide helpful insights for others developing digital interventions that include human support.

### Objectives

National guidance recommends conducting process evaluations to identify how new interventions are implemented in practice, the likely mechanisms through which they might produce an effect, or factors in the health care environment that might stop an intervention from producing an effect [17]. This paper reports a process study exploring HCPs’ perceptions of *Renewed*. Although the RCT of *Renewed* [13] is ongoing, as recommended by the Medical Research Council guidelines, qualitative process data are reported here before obtaining knowledge of the RCT outcomes to avoid biased interpretation [17]. This process study has been used to explore potential barriers to and facilitators of implementing *Renewed* in primary care and evaluate the acceptability of providing this type of support, which might contribute to the success (or not) of the intervention. Specifically, this study aims to explore (1) supporters’ experiences of providing support to patients using the *Renewed Online* digital intervention (from hereon referred to as *Renewed Online*) and (2) barriers to and enablers of the successful implementation of *Renewed Online* in practice.

## Methods

### Study Design

The study design entailed a qualitative process evaluation of the *Renewed* intervention, which explored HCPs’ perceptions of delivering support alongside *Renewed Online*. The COREQ (Consolidated criteria for Reporting Qualitative studies) checklist [18] guided the reporting (Multimedia Appendix 1 [18]). Participants in the RCT were randomized to (1) *Renewed Online*, (2) *Renewed Online* with brief human (HCP) support, or (3) usual care. For full details of the *Renewed* RCT, see the study by Krusche et al [13]. Briefly, survivors of cancer in the *Renewed* RCT (n=2712) had completed treatment for colon cancer (432/2712, 15.93%), breast cancer (1216/2712, 44.84%), or prostate cancer (864/2712, 31.86%). Mean years since the completion of treatment was 4 (SD 3.1) years; mean age was 64.5 (SD 10.9) years; and mean baseline QoL score was 72.4 (SD 11.9; as defined by scores <85 on the European Organization for Research and Treatment of Cancer measure [19]).

### Ethics Approval

Ethical approval was granted by the University of Southampton (ERGO reference 31000.A8) and National Health Service (reference 18/NW/0013) ethics committees.

### The Renewed Intervention

#### Overview

**Figure 1:**
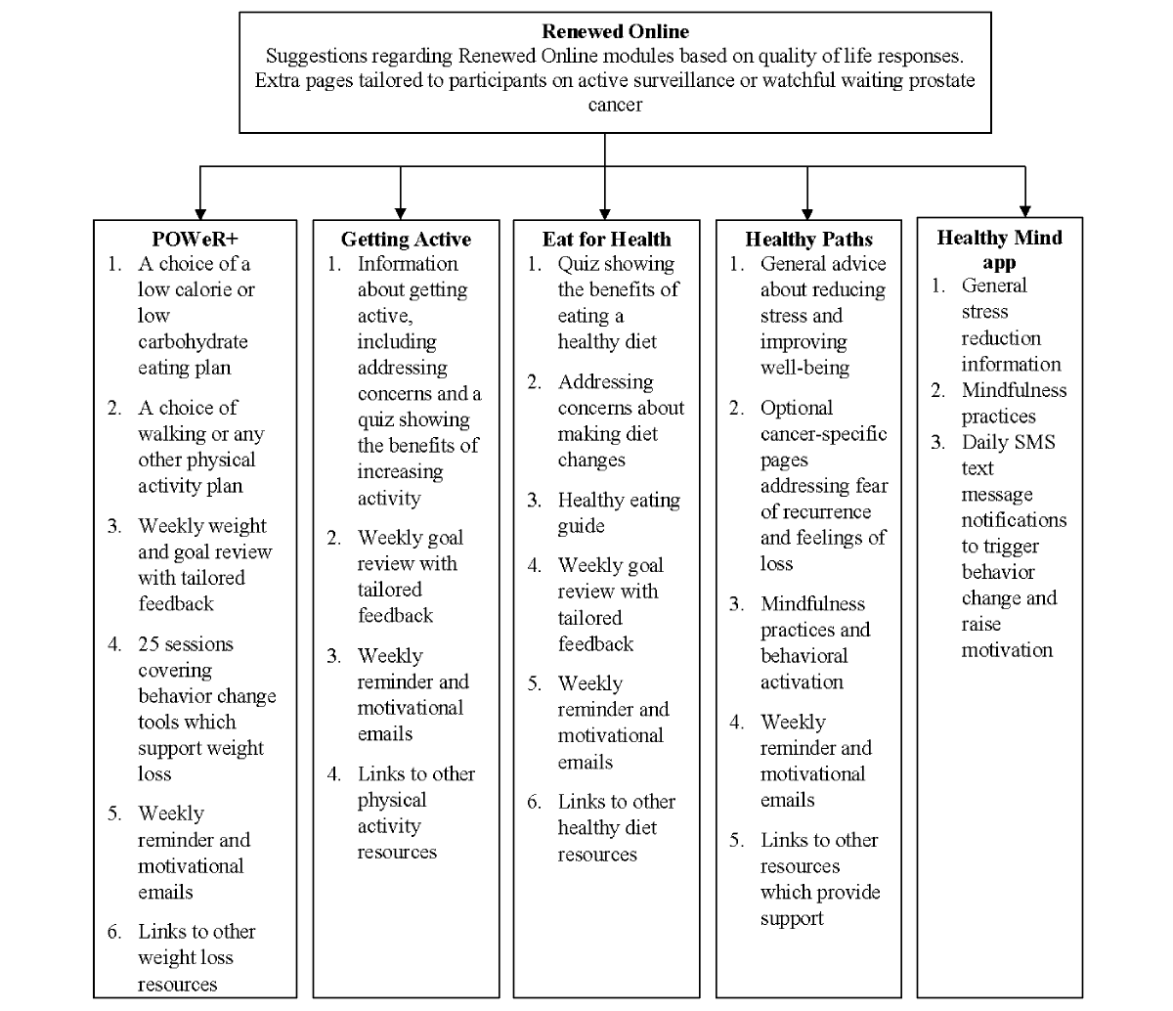
Renewed Online intervention (reproduced with permission from Krusche et al [13]).

Renewed comprises a component website, *Renewed Online*, and brief HCP support. *Renewed Online* comprises an introductory session that provides an overview of what to expect from *Renewed*, brief advice on how to treat symptoms, and tailored recommendations about which components of the program would be most helpful based on the users’ responses to the European Organization for Research and Treatment of Cancer measure [19]. Users can then choose to use *Getting Active* (support for increasing physical activity), *Eat for Health* (support with healthy eating), *POWeR* (an evidence-based weight loss program [11,20-23]), or *Healthy Paths* (support with reducing stress or difficult feelings [24]). A full description of *Renewed Online* is provided in Figure 1 [13], incorporating the TiDIER (Template for Intervention Description and Replication) guidelines (Multimedia Appendix 2) [25].

#### HCP Support

HCP support was designed to boost adherence to both using the website and engaging with offline behavior changes (eg, physical activity) by promoting autonomous motivation. Survivors of cancer allocated to the *Renewed Online with brief human support group* were able to access support sessions provided by an HCP, delivered using the *congratulate, ask, reassure, and encourage (CARE)* approach [26]. CARE is based on the self-determination theory and aims to facilitate an autonomy-supportive relationship that promotes feelings of autonomy, competence, and relatedness [21], thus building internal motivation for change [27]. CARE was designed to be easy to deliver and fit within HCPs’ busy schedules, without practitioners needing to become experts in a particular condition or way of treating that condition as this more detailed behavioral support was instead provided by the website.

Supporters were practice nurses, practice-based health care assistants, or clinical research nurses who were part of a comprehensive research network outside of general practitioner (GP) practices, a model representing delivery of care similar to that provided by private companies supporting digital interventions in the NHS, who tend to provide phone rather than in-person support and do not have access to patient records [28]. At the start of the study, supporters completed brief 15- to 20-minute web-based training outlining the study procedures and how to provide support to patients using the CARE approach. Before the sessions, the supporters were asked to send emails to patients 2 and 4 weeks after the patients began the study. Friendly email templates were provided, which were framed around the CARE approach, asking how patients were getting on and encouraging them to get in touch for a support session if they wished. Support sessions of 10 minutes were offered 2, 4, and 8 weeks after the patients had begun the study via telephone, email, or face to face. Textbox 1 shows a brief summary of the key messages from supporter training on how to provide support.

**Supporter training key messages.**

**Brief summary of the guidance given to supporters on how to provide support**

**Use the congratulate, ask, reassure, encourage approach with patients during support sessions**

- Congratulate the patient; for example, “That’s great that you want to get more active”
- Ask the patient; for example, “Have you decided to make any of the changes that Renewed suggested might be helpful?”
- Reassure the patient; for example, “Yes, doing more physical activity is safe and should help you to feel better.”
- Encourage the patient; for example, “Keep going with that as it should start to help you to feel better soon.”

**Tips for providing support**

- Be warm and friendly
- Praise any achievements
- Listen and show understanding

**When sessions should take place**

- 2, 4, and 8 weeks after the patient signs up for *Renewed*
- Send an encouraging email at 2 and 4 weeks using the supporter website; editable prewritten email templates are available
- Log all emails and appointments on the support log

**If a patient does not contact for support**

- Send an encouraging email

**If you find it hard to talk to the patient for only 10 minutes**

- Start the session by saying, “Nice to speak to you today. This is just a short appointment, we have around 10 minutes to talk. It would be great to hear how you’re getting on with Renewed.”
- In the last few minutes, say, “We are coming to towards the end of our time, is there anything else that you wanted to discuss quickly today?”
- Let the patient know that the session is about to end; say, “Thank you for your time, it’s been nice to chat with you”

**If the patient asks for advice**

- Ask them what they think would work best for them or what they think would be best to do.
- It is okay to ask, “what does the website say to do in that situation?”
- If the patient is concerned about whether making a change is safe, you can reassure them that everything recommended on *Renewed* is safe.

### Sampling and Recruitment

Supporters were identified for interviews through the *Renewed* supporter database and the study team’s records of HCPs providing support as part of the RCT. Emails or phone calls were used to invite supporters to participate in a telephone interview about their experience of supporting patients using *Renewed Online*. In the early stages of recruitment, supporters were sampled purposively based on their job roles (practice nurse, practice-based health care assistant, or clinical research nurse); however, recruited supporters often had not undertaken any support sessions or only supported 1 patient. Supporters were then purposively sampled based on the number of patients they had supported to ensure the inclusion of those who had supported multiple patients to explore any variation in their experiences. Supporters were provided with a participant information sheet and asked to confirm their informed consent on the web after consideration.

### Procedure

Interviews were conducted between September 2019 and January 2020, each lasting approximately 15 to 30 minutes, with a median of 21 minutes. A total of 2 (JS and JSB) researchers conducted the interviews. A semistructured interview schedule was developed by a qualitative researcher (JS) and experienced health psychologist (KB). The interview schedule explored supporters’ experiences of providing support along with the digital intervention, perceptions of web-based supporter training, experiences of support appointments, perceptions of the CARE approach, and supporters’ perceptions of the *Renewed* program.

### Data Analysis

All interviews were audio recorded, transcribed verbatim, and then imported into NVivo 12 (QSR International) [29]. An inductive thematic analysis was performed based on aspects from the 6-step framework of Braun and Clark [30] and Joffe and Yardley [31]. JS familiarized herself with the data before coding the interviews. A coding manual was created and continually updated to reflect the ongoing analysis. The identification and validation of the developing themes were achieved through an iterative data analysis process with frequent discussions with KB, RE, and AR. Deviant cases were considered to ensure that minority views were not overlooked [32]. An audit trail and reflective log were completed to maintain rigor during the analysis. Constant comparison (a technique in which each interpretation and finding is compared with existing findings as it develops from data analysis [33]) was used to examine potential similarities or differences in the reported experiences of different types of supporters [34].

## Results

### Participant Characteristics

A total of 108 supporters were invited to participate in the interview, of whom 56 (51.9%) did not reply to invitations, 21 (19.4%) could not be interviewed as they had not undertaken any support sessions, 2 (1.9%) did not have the time to take part in an interview, and 1 (0.9%) could not accurately recall supporting patients. The final sample included 28 HCPs comprising 16 (57%) practice nurses, 6 (21%) clinical research nurses, and 6 practice-based health care assistants (21%) who provided support for patients at 45 GP practices in total. Almost all participants were female (27/28, 96%).

### Themes

#### Overview

A total of 4 themes were developed that provided insights into supporters’ experiences of providing support along with digital interventions and factors that hindered or enabled them to support patients as intended. The themes were (1) *Renewed Online* as an acceptable digital tool with some improvements, (2) confidence in enacting the supporter role, (3) practicalities of delivering support alongside a digital intervention, and (4) managing a patient-led approach. Each theme is outlined in the following sections, including representative quotes to illustrate key points. Participants are referred to by their identification number, role, and the number of patients they supported.

#### Renewed Online as an Acceptable Digital Tool With Some Improvements

Overall, supporters perceived *Renewed* as consistent with current practice, with the increasing use of web-based interventions. They could see how a digital tool such as *Renewed Online* would be useful for patients, especially as it allowed patients to work through rehabilitation at their own pace:

They’re [GPs] signposting patients to online resources all the time more and more at the moment...So this [Renewed Online] is a similar thing. So I could see that it would be beneficial and would fit in.

Participant 10, practice nurse, 2 patients

Email support was also generally acceptable to supporters. However, a few worried that patients were not receiving emails from the supporter website; hence, they preferred to use their own email to contact patients.

A minority of supporters reported that their patients described the content of the information on the *Renewed Online* website as generic, not personal, and failing to provide anything new. These patients chose not to be part of the program:

He felt that the website was very generic and wasn’t personal to him. He was like, “I already know all of that.” he felt that it couldn’t offer him any support at all...I couldn’t then offer him any support with anything because he didn’t want it. He said, “If you could give me advice on specific areas,” which obviously we couldn’t do.

Participant 23, practice nurse, 1 patient

Approximately 7% (2/28) of supporters raised concerns over the timing of providing *Renewed Online*. They suggested that it was important for *Renewed* to be introduced to patients when they first finish treatment and support from the hospital ends. At that point, they felt that *Renewed Online* could better support them and be more of a teachable moment before patients form their own habits for managing side effects or returning to old ones:

What would be brilliant, would be to get it in...very soon after they’ve finished their final treatment...because that’s when they’re perhaps the most vulnerable...giving them a tool where they can work out what’s gonna benefit them in their life at that point. I think two, three years down the line, however they’ve got there, they’ve got there on their own without that [Renewed] kind of support.

Participant 15, practice nurse, 4 patients

#### Confidence in Enacting the Supporter Role

Supporters received web-based training at the start of the study on how to provide support alongside digital interventions (Textbox 1). This explained how to use the CARE approach to support patients’ engagement with *Renewed Online* and emphasized that the supporter did not need to be an expert in cancer. Most supporters reported that the length of training was adequate and provided clarity on what was needed for the role:

It was thorough, it explained everything really well I wasn’t left with any questions. It was clear and easy to follow.

Participant 13, clinical research nurse, 1 patient

Some supporters possessed prior experience in cancer care and expressed confidence in their role supporting *Renewed Online*. Although not previously experienced in this area, others still expressed confidence but reported that this had grown as they gained experience in delivering the intervention. Although there appeared to be little substantive differences in the experiences of HCPs who supported multiple patients compared with 1 patient, the associated greater frequency of delivering support appeared to allow HCPs more opportunities to build confidence:

The more you do the calls, or the email correspondence...the much easier I feel it’s become.

Participant 1, clinical research nurse, 3 patients

On the other hand, deviant case analysis highlighted that 33% (2/6) of health care assistants were the only supporters to report an initial lack of confidence based on preheld perceptions that they were unqualified for the supporter role. The first (participant 5, 2 patients) reported that the training did not prepare her for the role, expressing a lack of understanding of how to provide support and wanting to receive practical demonstrations of someone providing support. The second doubted her suitability for the role, initially being concerned that she was not an expert in cancer. However, these perceptions changed, and their confidence appeared to grow when actually delivering sessions, demonstrating that their initial concerns were perhaps unwarranted:

I felt like a bit of a fraud at the beginning, thinking am I really qualified to do this, I feel like the patient’s phoning me up thinking I’m some sort of expert, but it wasn’t like that at all.

Participant 17, health care assistant, 2 patients

Differences in where the supporters were based (either practice based or remote in the case of clinical research nurses) appeared important to their experiences in supporting patients. In particular, a few clinical research nurses felt disadvantaged based on the assumption that practice staff were probably more familiar with patients. They felt that this would facilitate rapport with patients and improve the quality of the support sessions:

It [Supporter role] would need to be somebody from the practice actually doing it who has access to their medical notes...just so that you’re aware when you’re listening to them, so you know what they’re going through rather than being completely blind.

Participant 8, clinical research nurse, 3 patients

#### Practicalities of Delivering Support Alongside a Digital Intervention

Reflected in this theme is an exploration of the logistical problems supporters faced while delivering support to patients using *Renewed Online*.

Most of the current sample expressed difficulty in conducting sessions in the recommended 10 minutes, often reporting sessions of approximately 15 minutes. Sessions lasted >10 minutes for various reported reasons, including allowing time for introductions, the perception that patients felt lonely and were longing for someone to talk to, and not wanting the patient to feel rushed. In particular, the primary care staff expressed guilt about potentially rushing patients, considering that they had made an effort to come in for sessions. A clinical research nurse expressed difficulty in managing the 10-minute sessions as she was not used to working within this time limit:

I’d given myself longer than what was suggested because I knew from experience that if somebody is opening up to you about how they’re feeling the worst possible thing that you can do is run out of time and have to end it.

Participant 24, practice nurse, 2 patients

A few supporters expressed a preference for lengthening sessions, particularly the first, to allow more time to get to know the patients and address any initial concerns. Relatedly, some clinical research nurses reported finding it challenging to build rapport with patients during the brief support sessions:

The appointment seemed very short. Especially on your initial one. I think your initial appointment should be twenty. So you can get to know the patient a bit before you bang straight into the CARE approach. Otherwise there’s no real time to even introduce myself, introduce themselves.

Participant 23, practice nurse, 1 patient

HCPs viewed both face-to-face and telephone support as acceptable but with different benefits. Face-to-face sessions allowed them to read the patients’ body language, whereas phone support was better for patients who may have difficulty in coming into a GP surgery because of travel disruptions, weather conditions, and location. In addition, phone sessions provided greater flexibility to supporters as it was easier to slot into their schedules:

That [phone sessions] works really well for me because it means that I can support patients when I’m not in the office...that’s given me a greater flexibility with the patients.

Participant 2, clinical research nurse, 5 patients

Furthermore, phone sessions reportedly helped some supporters manage the length of sessions by preventing them from performing health care checks unrelated to *Renewed*. Supporters also expressed less *guilt* of having patients make the journey into practice.

#### Managing a Patient-Led Approach

Reflected in this theme were supporters’ perceptions and experiences of using a patient-led approach and what they saw as helpful and found difficult. In this context, a patient-led approach refers to one in which an autonomy-supportive relationship was facilitated using CARE to support the digital intervention rather than giving advice, which was instead provided through the digital intervention. Most supporters reported that they liked the CARE approach and believed that it provided a useful prompt and session guide:

I liked that idea [CARE approach]. I thought that was really well planned and it’s easy to remember...a good thing to just prompt you.

Participant 26, practice nurse, 1 patient

During sessions, patients would often discuss their behavior change goals and progress. Supporters expressed that it was initially a challenge not to give direct advice to patients during sessions. However, this reportedly became easier as they delivered more appointments. One of the supporters expressed that it was nice to see patients who were actively interested in improving their health:

It was refreshing to see them wanting to make life changes themselves rather than making lifestyle changes because they’d been advised to by a clinician.

Participant 24, practice nurse, 2 patients

In addition, some supporters expressed that not giving direct advice was a positive change and welcomed patients being more involved in their care:

It’s all about them giving us the answers as opposed to the other way round, which I’m all for. I think that’s better.

Participant 23, practice nurse, 1 patient

A few supporters’ experiences portrayed a lack of understanding of the CARE approach and how to implement it, which caused some difficulty in delivering support alongside the digital intervention. For example, one of the supporters found it challenging to implement this approach when the patients went off on a tangent. She believed that this was because she viewed the CARE approach as a *script* to be followed strictly in a specific order, which made the conversation rigid:

I think that’s why sometimes I didn’t manage to get the CARE aspects in the way I’d like because sometimes you would start at one element of it, and you think, “Okay, I must make sure I go back to the C element or the A element...” And then I’d be like, “Well, how do I sort of interject that in now? Now we’re kind of talking about something slightly different.” I wanted it to more fluid.

Participant 12, clinical research nurse, 1 patient

This supporter viewing CARE as a script may reflect a more traditional understanding of HCP-patient relationships in which HCPs provide systematic education and instruction. However, CARE encourages an approach that prompts supporters to help patients decide what works best for them, perhaps indicating the supporter’s misunderstanding or lack of familiarity with the CARE approach.

Relatedly, a practice nurse doubted the CARE approach as she perceived that patients wanted direct advice from her rather than just the website. Consequently, she felt quite limited in her supporter role.

Approximately 7% (2/28) of supporters highlighted that they would have liked to be able to review patients’ *Renewed Online* activity so that they could be aware of what patients were referring to during appointments:

They would talk to me and I’m not completely sure I knew everything that they were covering [Renewed Online activity]...So that’s something that I found difficult because they would talk away as if I knew what they were talking about.

Participant 8, clinical research nurse, 3 patients

Other supporters printed off pages from the *Renewed Online* demo and brought them into support sessions to overcome this.

## Discussion

### Principal Findings

This process evaluation used qualitative interviews to understand supporters’ experiences of providing support to survivors of cancer alongside a digital intervention in primary care. Exploring supporters’ experiences enabled the identification of possible factors that hindered or enabled support being delivered as intended alongside a digital intervention, highlighting lessons for future intervention development and implementation. Overall, supporters felt that they were able to follow the protocol and deliver support as needed; however, several issues were identified that might hamper implementation, and some minor alterations to *Renewed Online* would likely be required to ensure that the intervention is optimized for successful implementation in practice. Considering implementation theory in process evaluations can provide a framework for evaluating and explaining the success of implementation [35]. Therefore, the findings will be discussed in relation to the normalization process theory (NPT) [36], an implementation theory that explains the processes through which new practices of thinking, enacting, and organizing work are operationalized in health care [37]. An outline of the NPT, as described by McEvoy et al [38], is provided in Textbox 2.

The aspects of the intervention that supported implementation included the ease of training and the perceived similarity of *Renewed Online* to digital tools used in current practice. In relation to NPT, this demonstrates a high degree of *coherence* regarding the value of *Renewed Online*, which is needed for an intervention to be successfully implemented well in practice. Positive perceptions of the utility of an intervention have been shown to be key facilitators of implementation [39], and implementation failure occurred when HCPs did not perceive intervention use as a legitimate activity for patients or providers [40]. Previous literature has suggested that HCPs in primary care may not be well placed to provide support to survivors of cancer as they lack the expertise and time necessary to make these changes and desire clearer guidance on how to do so [8,9]. However, this study found that primary care staff felt that supporting survivors of cancer by using a digital intervention would be appropriate and beneficial. It is possible that this finding differs from previous literature as this is the first study to explore the views of primary care staff providing support *alongside* a digital intervention. In most cases, this format seemed to overcome concerns about the lack of expertise and time, as the digital intervention provided specific advice, avoiding the need to develop expertise, and vastly reduced the amount of input needed to support survivors of cancer to make behavioral changes. A minority of supporters initially believed that their perceived lack of expertise would affect their ability to support patients. However, their confidence in this approach improved once they began to support the patients, suggesting that this was not a significant barrier to implementation.

Previous research on digital interventions for other conditions has shown that primary care staff have reservations about providing phone support, viewing it as less effective than face-to-face support [21]. The acceptability of phone support seen in this study may reflect the fact that primary care is changing and is increasingly using phone appointments to manage increasing workloads [41]. This may normalize more rapidly in the current climate, as telemedicine is increasingly advocated for use in those with cancer during the COVID-19 pandemic to minimize the number of visits to health care settings and risk of exposure [42]. This increase in acceptability has implications for the implementation of future digital interventions using primary care staff to support digital intervention users, as phone support may provide similar effects and be more cost-effective [20].

**Normalization process theory outline.**

**Construct and definition**

**Coherence**

The work individuals and organizations have to go through to understand a new practice to promote or inhibit it; these processes are energized by investments of meaning made by participants

**Cognitive participation**

The work individuals and organizations have to go through to enroll users and engage with a new practice; these processes are energized by investments of commitment made by participants

**Collective action**

The work individuals and organizations have to go through to enact a new practice; these processes are energized by investments of effort made by participants

**Reflexive monitoring**

The work of formal or informal appraising an intervention to develop participants’ comprehension of the effects of the intervention; these processes are energized by investments in the appraisal made by participants

Most supporters successfully engaged with the CARE approach, with some noting that not giving direct advice was a positive change and welcomed patients being more involved in their care. This provided evidence of both *cognitive participation* and *collective action* and suggested that for most supporters, the CARE approach would likely normalize well in practice. However, a minority experienced difficulty adjusting to providing nondirective support and instead allowing the digital intervention to provide the advice. In terms of NPT, there was an apparent lack of *cognitive participation,* which suggests a potential challenge for successful implementation. In the wider literature, HCPs’ difficulty in adjusting to not giving direct advice is a prevalent pattern. Encouraging health care workers to switch from a more traditional paternalistic approach, in which they hold all the knowledge and power and give it to the patient, to an equal relationship using nondirective support often requires intensive training, including reflective practices [43,44]. This is an issue that is pertinent to providing human support alongside many digital interventions, where health care workers are often employed to boost engagement but are not expected to be experts or to give advice [20,26]. It is possible that more intensive training might help the minority who struggle with the CARE approach. Alternatively, it may be that employing staff specifically to provide this support is more feasible than implementing more intensive training to change the behavior of health care workers whose daily work usually involves working in a directive way (eg, giving advice). Such an approach has been adopted successfully in a digital diabetes prevention program in which a commercial company (Changing Health) provides telephone support to NHS patients using digital services [28].

Some clinical research nurses perceived that not being based within GP practices was a barrier to delivering support as intended, as they did not have a pre-existing relationship with patients or access to their medical records and consequently reported finding it challenging to build rapport during 10-minute sessions. NPT would see this as a challenge to *collective action,* which examines the work HCPs have to do to enact a process [36]. This is an important issue, as the model of using research nurses adopted in this study is similar to that adopted within health care elsewhere, such as when private companies provide telephone support alongside digital interventions to patients in the United Kingdom’s NHS (eg, the NHS digital diabetes prevention program); these workers do not have prior relationships with patients or access to their medical records. It may be that within such a context, a longer (perhaps double) appointment is needed to provide time to build rapport, as rapport building is considered crucial to quality health care support [41].

Some supporters suggested that *Renewed Online* should be offered to patients sooner after finishing treatment as this may be when patients are most vulnerable and motivated for behavior change. This demonstrates the NPT construct of *reflective monitoring*, whereby supporters’ appraisal of *Renewed Online* considered the potential disadvantages and suggested how implementation may be improved in the future. In line with supporters’ suggestions, previous research found that survivors of cancer described feeling the drive to adopt a healthier lifestyle to feel better and more empowered immediately after finishing treatment, and hence, it may be that this is the optimal *teachable moment* [15].

In light of the experiences of supporters and the barriers identified, several issues were identified, and potential plans for addressing these issues are presented in Table 1.

**Table 1:** Plans for addressing challenges faced by supporters.

Challenges faced by supporters	Plans for addressing those challenges
Many supporters were concerned that the 10-minute support sessions were too short.	Giving the option for the first session to be a double appointment should allow the time for initial introductions and addressing concerns.
Some clinical research nurses perceived that not knowing the patient before the first session was a disadvantage, as they had no existing rapport to build on.	Having the first session be an optional double appointment should allow time to build more rapport before beginning support.
Some HCPs^a^ expressed a desire to see patients’ activity on *Renewed* to enable easier and most salient conversations during sessions.	It may be useful to provide supporters with access to patients’ *Renewed* activity.
Supporters suggested *Renewed* should be introduced at the point when patients are leaving cancer treatment as this is potentially when they are most in need of support.	Future implementation of *Renewed* may need to concentrate on patients who have finished treatment more recently instead of up to 10 years after treatment.
A few supporters were reluctant to use the CARE^b^ approach as it was different from a traditional health care worker–patient relationship where the HCP is seen as having control and provides advice.	Training could be intensified for the minority who have concerns about not giving advice. This could include reflective practices, which have been shown to help people switch from a directive to nondirective approach [43,44].
A few supporters expressed a misunderstanding of how to use the CARE approach.	Update supporter training to include video demonstrations of how CARE can be delivered.
Some supporters expressed that delivering more support enabled them to build confidence.	Have fewer supporters so that they are able to support a greater number of patients, which could give them the opportunity to build confidence in delivering support.

^a^HCP: health care professional.

^b^CARE: congratulate, ask, reassure, and encourage.

### Strengths and Limitations

The variation in HCP roles included in the study allowed the nuanced experience of those in different job roles to be explored. This study has several limitations. First, the data could not be analyzed iteratively during the interview period. This meant that the themes developed in early interviews could not be explored further in later ones, which can develop meaning and understanding [45]. Second, most (401/557, 71.9%) logged support sessions in the Renewed RCT were reported as sticking to 10 minutes within support sessions; however, those who consented to the interview gave patients 15 minutes on average within support sessions. It is difficult to know why this study’s sample differs from the overall trial sample in this way and whether it might limit the transferability of results. This difference may be because of the use of paper self-report measures to collect the duration of support sessions within the trial, possibly resulting in a social desirability bias [46]. However, given the opportunity in an interview to discuss this in more detail, HCPs may have been more inclined to mention if they went over 10 minutes and why. Third, we were unable to record consultations with supporters within this study; hence, we could not corroborate supporters’ reports on how they implemented the CARE approach. Further research exploring the recorded consultations of supporters using CARE would be useful. Finally, there was a low response rate to the interview invitations. There may be various reasons for such a low response, one of which may be the capacity for HCPs to conduct interviews because of busy schedules. The perceptions and experiences of implementing support alongside *Renewed* may have differed for those who did not accept an invitation to interview.

### Conclusions

Our results suggest that HCPs generally found providing support alongside a digital intervention acceptable and were amenable to contributing to the delivery of support to survivors of cancer in primary care. Key factors that may support the successful implementation of this type of digital intervention in practice include the increasing acceptability of phone support and the utility and acceptability of nondirective support among most HCPs, such as the CARE approach. Challenges to implementing support alongside a digital intervention were also identified, including concerns about not having enough time during support sessions to build rapport and, in a minority, concerns about using a nondirective approach. This study shows that even when support for a digital intervention is designed to be brief, sufficient time needs to be allowed in the initial support sessions to allow practitioners to feel confident that rapport can be built. Further research is needed to explore whether additional training might be enough to support a minority of health care practitioners who were concerned about giving nondirective support to adopt this approach. If not, then primary care could consider employing other staff, such as social prescribers of health coaches, who work in a less directive way than nurses and health care assistants and who are now becoming increasingly common in the United Kingdom’s NHS [47].

There is a clear need for primary care to provide support to survivors of cancer [7]; however, previous research has suggested that lack of time and training on how to support this patient group are key barriers to providing this support [8,9]. This study showed that providing support alongside a digital intervention might be an acceptable way of overcoming these barriers, as only a small amount of support is required, and there is no need to develop cancer-specific expertise or behavior change skills. This approach of mixing digital and human support will likely be useful to others in developing and implementing interventions to support other aspects of care for survivors of cancer, which are not targeted within *Renewed Online*, such as support for sexual dysfunction, smoking cessation, alcohol consumption, returning to work, and lack of social connection and support.

## Appendix

Multimedia Appendix 1 COREQ (Consolidated Criteria for Reporting Qualitative studies): a 32-item checklist.

Multimedia Appendix 2 The TiDIER (Template for Intervention Description and Replication) checklist.

## Abbreviations

CARE: congratulate, ask, reassure, and encourage
COREQ: Consolidated Criteria for Reporting Qualitative studies
GP: general practitioner
HCP: health care professional
NHS: National Health Service
NIHR: National Institute for Health Research
NPT: normalization process theory
QoL: quality of life
RCT: randomized controlled trial
TiDIER: Template for Intervention Description and Replication
